# Teleconsultation in health and social care professions education: A systematic review

**DOI:** 10.1111/tct.13519

**Published:** 2022-07-27

**Authors:** Lisa‐Christin Wetzlmair, Veronica O'Carroll, Andrew S. O'Malley, Stuart Murray

**Affiliations:** ^1^ University of St Andrews, School of Medicine St Andrews UK

## Abstract

**Introduction:**

Teleconsultation education in health care and social work education is under‐reported. However, literature indicates that educating the workforce in teleconsultation skills is essential to continue with safe, high‐quality delivery of services and increases the likelihood of implementing teleconsultations in health care. Training for students should, therefore, be encouraged. This systematic literature review aims to investigate global experiences of teleconsultation training in undergraduate health care and social work education.

**Methods:**

A systematic review of peer‐reviewed literature was undertaken. The review was guided by the Joanna Briggs Institute guidelines. Electronic databases were searched for eligible evidence. Studies were included only if they described and evaluated teleconsultation education for undergraduate health care and social work students.

**Results/Discussion:**

This review shows that mandatory education in teleconsultation is limited in undergraduate health care and social work education. Narrative synthesis and analysis of 14 studies led to the development of two themes: pedagogical aspects, and perspectives on telecommunication and teleconsultation learning and teaching. Practical experiences with simulated patients or during clinical placements with real patients were the most common mode of delivery. Feedback on teleconsultation education was generally positive; overall, health care students felt more confident using teleconsultation and valued safety of learning through simulation.

**Conclusion:**

Teleconsultation education is a legitimate way to expose students to telehealth. High satisfaction rates, increased knowledge and confidence in use indicate the positive impact this learning has on students. Nevertheless, further high‐quality research and guidance for educators are warranted.

## 1 INTRODUCTION

Teleconsultation is defined as a consultation provided remotely by using information communication technology (ICT) such as telephone and videocommunication platforms.^1^ Despite evidence highlighting that teleconsultations are as effective as face‐to‐face interventions in terms of health outcomes and costs,^2^ further endeavours to develop and strategically implement digital technologies in health care systems are needed.^3^

Literature suggests that teleconsultations are well established in care and management of long‐term conditions. Nonetheless, the onset of the COVID‐19 pandemic led to a surging number of teleconsultations via video or telephone worldwide.^4^, ^5^ National guidelines have been developed to acknowledge the way in which communication with patients has changed.^6^ This guidance is necessary as teleconsultations require different skills in digital communication.^7^ Teleconsultations must, therefore, be addressed in education and training for health care and social work professionals and students, in the same way that face‐to‐face communication is.^6^, ^7^

The Topol Review^8^ briefly acknowledged the need for training future health care workers and predicted that remote consultations would replace most of the face‐to‐face interactions in the future. Educational initiatives for health care and social work professionals are, however, under‐reported.^9^ Furthermore, although universities are responsible for preparing the future health care workforce for a digitalised health care setting,^10^ the guidance for delivering this education, especially at undergraduate level, is limited,^11^, ^12^ focussed on training at post‐graduate level, or is geographically limited.^9^, ^13^ This limited knowledge and understanding of teleconsultation training in undergraduate education worldwide highlights the need for teleconsultation training in undergraduate education.^14^

Limited knowledge and understanding of teleconsultation training in undergraduate education worldwide highlights the need for teleconsultation training in undergraduate education.

This systematic review aims to describe how teleconsultation learning and teaching is delivered to undergraduate health care and social work students in academic and clinical settings and to explore how teleconsultation learning and teaching is received by students and faculty.

The initial question guiding this review is: *How is teleconsultation experienced and taught (delivered, implemented, assessed) in undergraduate health care and social work education?*

## 2 METHODS

This mixed‐methods systematic literature review strategy was based on the Joanna Briggs Institute (JBI) guidelines,^14^ and the reporting followed the PRISMA Statement (Supporting Information C).

Guided by the SPICE framework,^15^ key terms were identified, and a search was conducted using the databases displayed in Table 1. Key terms were adapted for the requirements of each database. An example PubMed search strategy is provided in Supporting Information A.

**TABLE 1:** Searched databases, the SPICE framework and related key words for the search strategy

Databases
Academic Search Complete, British Education Index, Educational Administration Abstracts, ERIC and MEDLINE (accessed through EBSCOhost), CINAHL, Embase, PubMed, Scopus and Web of Science.

	SPICE component	Key terms		
S	Setting *Where?*	Higher education, academic, placement, undergraduate stud*, college, institute, center/centre		
P	Perspective *For whom?*	Undergraduate med* student Preregistered med* student Prequalified med* student	health professional student*, allied health student* nursing students	Social work student*
		teachers, professors, trainers, educators, tutors, facilitators, faculty		
I	Intervention *What?*	curriculum, learning, teaching, education		
		Telehealth, teleconsultation, telemedicine, video consultation, telecommunication, information communication technology, digital health, e‐health		
C	Comparison *Compared with what?*	face to face communication, face to face consultation, in person communication, in person consultation		
E	Evaluation *With what result?*	Skill acquisition, skill development, knowledge acquisition, knowledge development eHealth literacy		
		attitude, experience, perceptions, feedback, recommendations		

The results of all searches were collected, uploaded to and stored in the literature management software EndNote. Three authors (AOM, LCW and VOC) independently screened the literature against the a priori defined eligibility criteria (Supporting Information D) and discussed below. Disagreements were resolved through discussion among the authors. Quality appraisal was undertaken using the JBI appraisal checklists (Supporting Information B).

To find underlying themes and concepts across the included studies, a thematic synthesis approach^16^ was used. Quantitative and qualitative data from each study were compared, contrasted and summarised qualitatively.

## 3 RESULTS

**FIGURE 1:**
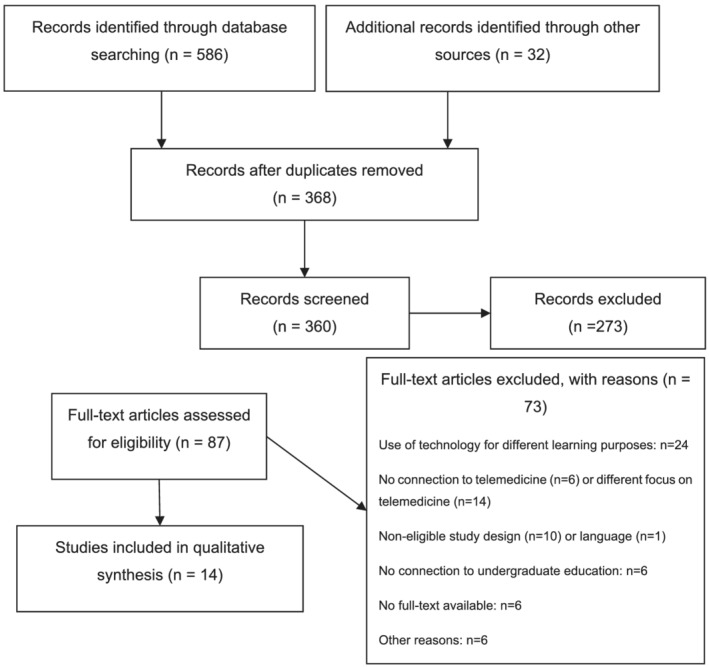
PRISMA flow chart: Eligibility criteria were applied at all stages of article screening and assessment. Full texts were excluded with reasons

A total of 586 records were identified by the database search. An additional 32 records were identified from screening the reference list of relevant papers. After removing duplicate records, the title and abstract of 360 records were screened, which resulted in 87 studies being identified for full‐text evaluation. Of these, a total of 14 studies matched the eligibility criteria (Figure 1).

The study characteristics and main results are summarised in Table 2. Eight studies were conducted in the USA,^17^, ^18^, ^19^, ^20^, ^21^, ^22^, ^23^, ^24^ two in Australia^25^, ^26^ and the UK^27^, ^28^ and one each in Germany^29^ and Spain.^30^ The studies were published in 12 different journals between 2010 and 2020.

**TABLE 2:** Study characteristics and main results. Critical appraisals for mixed‐methods studies mention the quantitative methods first. The results are displayed in the number of items answered with yes (Y)/no (N)/unsure (U)/not applicable (NA)

Author(s), year	Journal	Country	Study aim	Target group	n	Setting (mode, length)	Study type, type of evaluation	Results	Critical appraisal Y/N/U/NA
Abraham et al., 2020	Cureus	USA	To evaluate a telemedicine experience during the COVID‐19 pandemic	3rd year medical students	20	Theoretical online course and seminar, telephone screening with patients, shadowing teleconsultations between physicians and patients	Mixed methods	Learning about telemedicine would be an asset to students' education as students were not familiar with the concept of telemedicine. Students value the different patient scenarios and the immediate feedback from the practitioners after the consultation. This form of learning was an appreciated possibility for students to have contacts with patients during the pandemic.	1/5/0/2 0/10/0/0
Bulik & Shokar, 2010	Journal of Telemedicine and Telecare	USA	To report on an elective course on telemedicine	4th year medical students	7	Online introduction lectures, site visits and a reflective paper	Mixed methods	All students indicated that it was an effective, inspiring experience and would recommend it to their peers. After the training, students felt they acknowledge pitfalls in telemedicine more and they understood the opportunity for telemedicine for managing chronic diseases.	1/4/3/0 1/7/2/0
Cantone et al., 2019	MedEdPORTAL	USA	To expose students to telemedicine and assess videoconsultations	2nd, 3rd, 4th year medical students	287	TeleOSCE during students' clerkship	Qualitative study	The students felt the need for a training in teleconsultations before taking the teleOSCE; however, feedback was generally positive. However, students would prefer to be assessed on single skills rather than the combination of more skills. The immediate feedback after the teleOSCE was perceived useful.	1/7/2/0
Christner et al., 2010	Academic Medicine	USA	To assess the effects of training in email communication	Medical students	89	Group teaching session, including group discussions regarding email communication	Pre–post design	Knowledge about email communication, communication skills and professionalism improved significantly after the training.	4/4/0/0
Darnton et al., 2020	Medical Teacher	UK	To evaluate students' and supervisors' experience of clinical experience during the COVID‐19 lockdown measures	2nd year medical students	13	Teleconsultations supervised by practitioners, consulting from home	Qualitative study	The students were satisfied with the variety of patient cases encountered during the teleconsultations. The immediate feedback from the practitioners was helpful; however, the missing face‐to‐face interactions made the feedback for the supervisors more difficult. Choosing the appropriate patients for the teleconsultations was challenging for the practitioners. Students had to make effort to ensure patients' confidentiality and reported on technical difficulties as being stressful. The missing physical examination reduced the students' perception of educational value in terms of clinical skills.	6/0/4/0
Dzara et al., 2013	Academic Psychiatry	USA	To evaluate telepsychiatry training, the perception of supervision and the usefulness for future work	Medical students and residents	15	Students conducted teleconsultations and remote examinations while supervised by a clinician	Cross‐sectional study	The training was perceived as relevant for students' future career and enhanced the understanding of clinical responsibilities in telepsychiatry. The communication with patients was perceived more difficult. Even though the supervision was helpful, previous theoretical knowledge about telepsychiatry would have increased the learning outcome.	1/2/5/0
Edirippulige et al., 2012	Journal of Medical Internet Research	Australia	To assess students' perceptions of an eHealth practicum	Health science students	66	One day, theoretical lectures, practical activities, observations of telecommunications, visits to telehealth centres	Qualitative study	Most students rated the practicum as a relevant activity to gain practical skills in telemedicine for their future career. Students would prefer a stronger focus on high‐quality practical activities rather than lectures in the beginning, which were perceived as a repetition of components learnt in a previous eHealth course.	1/6/2/1
Fisher et al., 2014	Clinical Teacher	UK	To develop and evaluate a simulation training in interprofessional telephone communication	3rd year medical and surgery students	52	Previous communication training, lecture, simulation between students, nurses and practitioners	Mixed methods	The self‐rated confidence in interprofessional communication with nurses and physicians increased significantly after the simulation training. The safe learning environment was appreciated, and students wished to have more time to practise telecommunication with other professionals. The training they received prior was perceived insufficient.	2/4/1/2 0/8/2/0
Jimenez‐Rodriguez & Arrogante, 2020	Healthcare	Spain	To analyse the perception and satisfaction with video consulting simulation scenarios	3rd year nursing students	93	Three patient scenarios (lasting 4 hours each), online video conferences, small groups of 12 to 16 students	Mixed methods	Students were satisfied with the simulation scenario; the learning felt real, safe and relevant for their future career. All students agreed on the practical utility of the training regarding gaining clinical and communication skills. However, students did not think that technical skills improved. The opportunity to make consultations from home had a calming factor for students and they felt less nervous.	5/2/1/0 2/1/7/0
Mulcare et al., 2020	MedEdPortal	USA	To develop and assess a curriculum to practise telecommunication and teleconsultation communication strategies	Medical students	98	Eight‐hour programme for small groups (8 to 16 students), in‐person lecture, practical activities, online assignment	Mixed methods	Students rated their preparedness for teleconsultations higher after the training. Telemedicine was perceived as relevant for the future work. Especially the simulation scenarios were perceived useful; however, students wished for an earlier exposure in their education. The training should focus more on patient management rather than general information about telemedicine. Students wished for seeing and analysing examples of ‘real‐life’ teleconsultations by a physician or health care professionals.	1/2/3/3 2/6/2/0
Palmer et al., 2015	Rural Remote Health	USA	To assess the acceptability and feasibility of teleOSCE	Medical students	9	Participation in a teleOSCE	Qualitative study	Taking the teleOSCE was a valuable experience, that did not take long, and raised awareness for patients' health needs in rural areas. It was important that students were familiar with the technology to solve technical difficulties during the exam. The teleOSCE gave students a new understanding of what telemedicine is and how teleconsultations can be used.	2/7/1/0
Palmer et al., 2017	Journal of Family Medicine & Community Health	USA	To assess the knowledge of, confidence in and attitudes towards telemedicine	Medical students	172	TeleOSCE as one out of four OSCE that students completed during their clerkship	Pre–post design	Knowledge and confidence changed significantly between active and inactive groups. The increase in attitude was not significant.	4/3/2/0
Rienits et al., 2016	Clinical Teacher	Australia	To rate students' understanding of the issues and their confidence in conducting a teleconsultation	3rd year medical students	59	Two‐hour programme, theoretical in‐person lectures and simulated patient cases	Pre–post design	Students did not feel very confident in conducting teleconsultations before the training, even though they rated their knowledge about technology high. The training in medicolegal aspects of teleconsultations was perceived as most important.	1/6/2/0 0/10/0/0
Waschkau et al., 2020	Zeitschrift fur Evidenz, Fortbildung und Qualitat im Gesundheitswesen	Germany	To describe and evaluate a course about the digitalisation in health care in Germany	4th year medical students	60	Six 90‐minute courses, mandatory course, students could choose topics, in‐person lecture, group work	Cross‐sectional study	The session enabled students to have a positive attitude towards teleconsultation and telemedicine. Students were mostly concerned about and want to learn about the technological aspects in teleconsultations.	2/4/1/1 0/9/1/0

Out of the 14 studies identified, 12 studies included medical students.^17^, ^18^, ^19^, ^20^, ^21^, ^22^, ^23^, ^24^, ^26^, ^27^, ^28^, ^29^ One study included nursing students^30^ and one other included allied health professional students.^25^ None of the studies included social work students. Only one study interviewed educators.^27^ The experiences and opinions of telehealth and teleconsultation education were assessed via mixed‐methods study designs in seven studies.^17^, ^18^, ^22^, ^26^, ^28^, ^29^, ^30^ Qualitative research design was adopted by four studies.^19^, ^23^, ^25^, ^27^ Two studies evaluated students' experiences after a learning activity.^20^, ^21^

Two main themes were identified from the thematic synthesis: pedagogical aspects, and perspectives of teleconsultation teaching and learning. The former theme refers to various structures and characteristics in relation to how teleconsultation was delivered. The latter theme refers to students' and educators' subjective meaning of teleconsultation education and their individual experiences with and opinions of teleconsultation.

Two main themes were identified from the thematic synthesis: pedagogical aspects, and perspectives of teleconsultation teaching and learning.

### 3.1 Pedagogical aspects

Generally, teleconsultation courses were mostly optional or elective for students.^18^, ^19^ Only one study reported on mandatory training.^29^ Where interactive sessions enabling students to practise teleconsultation skills were described, these usually occurred in small groups of 8 to 16 students^22^, ^26^, ^30^ who were in some cases subdivided into groups of two to three students.^27^, ^30^

The teaching sessions varied in length and lasted between one and a half hours and 1 day.^17^, ^22^, ^25^, ^26^ Courses were also split up into two half days^22^ or six afternoon sessions.^29^ The learning outcomes for the courses focussed on knowing about the importance of telehealth,^22^ the limitations and possibilities of telehealth in general,^18^, ^25^, ^26^ knowing the differences between teleconsultations and face‐to‐face consultations and recognising the effect of telehealth on clinician–patient relationships.^18^, ^26^

Lectures were used at the beginning of a course to introduce the topic and to prepare students for practical sessions.^18^, ^22^, ^25^, ^26^, ^28^ The content usually included definitions of telehealth^17^, ^29^; information on resources, tools and technologies^18^, ^22^, ^26^; best practice examples^18^; and business‐related information such as reimbursement, data protection, privacy issues and medicolegal aspects.^18^, ^22^, ^26^ The length of the lectures varied between 30^22^ and 90 minutes.^17^

Lectures were used at the beginning of a course to introduce the topic and to prepare students for practical sessions.

Following the lectures, most students had the opportunity to apply their skills and knowledge in a practical setting. Various activities included role‐plays of telemedical encounters with patients^22^, ^25^, ^26^ or other health care professionals,^28^ observations of clinical teleconsultations^25^, ^26^ and site visits to hospitals and health institutions offering telehealth.^18^, ^25^ In two studies, students were exposed to real‐life teleconsultations with patients during their practice placements.^17^, ^27^

Medical students primarily received individual feedback following a teleconsultation activity.^17^, ^19^, ^23^, ^27^, ^28^ In some cases, feedback was provided to the whole group.^20^, ^22^ Clinical and communication skills and knowledge were assessed via a virtual objective structured clinical examination (OSCE), the so‐called teleOSCE.^19^, ^23^, ^24^ The assessment duration ranged between 11^24^ and 20 minutes.^23^ Approximately 5 minutes was allocated to the provision of feedback.^23^, ^24^ The patient cases included themes and topics previously covered in students' teaching.^20^, ^23^, ^28^, ^30^

The perceived preparedness for teleconsultations prior to explicit training was limited.^17^, ^22^ However, theoretical lectures, clinical and communication knowledge and skills acquired prior to teleconsultation courses positively influenced students' confidence levels,^25^ and knowing the technical equipment used in teleconsultations was reassuring to students.^23^ Exposing medical students to simulated interprofessional collaboration and communication scenarios increased self‐rated confidence levels, and more interprofessional telecommunication training was requested.^28^

The perceived preparedness for teleconsultations prior to explicit training was limited.

### 3.2 Perspectives of teleconsultation learning and teaching

Students reported positive attitudes and were generally satisfied with their teleconsultation learning experiences, which were described as relevant for future work settings,^17^, ^21^, ^22^, ^24^, ^25^, ^29^, ^30^ beneficial, cool, effective, enjoyable, great, informative, inspiring, interesting, useful and valuable.^17^, ^18^, ^22^, ^23^, ^25^ Students reported that the scenarios felt realistic^28^, ^30^ and would recommend the elective course to their friends.^18^ Prior negative attitudes towards telehealth changed to positive attitudes following a teleconsultation course.^19^, ^29^ Overall, students emphasised that they would use telehealth in their future practice^23^, ^25^; however, students reported that more training would be helpful to fully understand the advantages of telecommunications and teleconsultations.^19^

Some students highlighted limitations of teleconsultations such as changes in communication and relationship with patients due to the lack of face‐to‐face interactions^19^ and missing cues in non‐verbal communication.^19^, ^27^ Further concerns were related to the limited possibilities for physical examinations, which caused a shift in the focus to communication and history taking skills.^27^ In one study, students received theoretical information on the possibilities and limitations of physical examinations.^17^ To practise physical examinations, students were asked to simulate a physical examination via video‐technology.^22^ Various other issues impacted on the confidence levels and the experience of students in conducting teleconsultations: encountering technical issues, the dependency on technology and good internet connection^27^, ^30^ and the relationships with patients.^27^ Generally, however, students preferred to focus on technological aspects in designated telehealth courses rather than combining skills with clinical and communication skills.^19^ Despite the positive impact of learning and teaching on telecommunication skills, practical experiences with ICT were not associated with an improvement in technical skills.^30^ In contrast, some students gained technological knowledge such as setting up a videoconference and taking clinical images.^25^

Students highlighted the need for teleconsultation training prior to the assessment as a first exposure to teleconsultations during the teleOSCE was perceived as stressful.^19^ Further training in telehealth before practical experiences^21^ and more teleconsultation best practice examples^22^ were regarded as valuable preparation.^24^

Generally, teleconsultation participation allowed students to practise communication skills. However, students also highlighted the importance of applying clinical reasoning, clinical knowledge and technological skills.^19^ Even though communication via ICT was reported to be challenging,^21^, ^27^ students perceived an improvement in clinical and communication skills after practising videoconsultations.^30^

Generally, teleconsultation participation allowed students to practise communication skills.

## 4 DISCUSSION

This systematic review aimed to describe how teleconsultation training in undergraduate health care and social work education is delivered, as well as to report on opinions and experiences associated with teleconsultation education.

In relation to how teleconsultation teaching was delivered, this review showed that it was often taught as an elective course rather than mandatory, meaning that students most interested in telehealth could pursue this as a specific learning opportunity. Mandatory training would ensure that all students have this learning opportunity and would highlight the importance of teleconsultation training within the undergraduate curriculum.

The main findings of this review were that there were a range of delivery methods including theoretical lectures, simulation‐based learning and experiences in placements. The most common method was simulation‐based learning, and few explored the students' experiences in placements. Simulation has been effectively used in health workers' education for several years,^31^, ^32^ and authentic, realistic case scenarios are appreciated and valued by students.^32^ In contrast, a study that evaluated students' learning after working with recorded real‐life general practitioner (GP) consultations found that students prefer to work with real patients rather than with simulated patients.^33^ This review highlights the need for further exploration of a range of methods to inform future evidence‐based teleconsultation education and the need for structured guidance for educators. The need for guidelines is reinforced by the desire and need of students to be educated on different technology applications and processes in the health care field (i.e., telehealth). This education should be considered, as telehealth is an emerging field.^1^

Although students' perspectives of the teleconsultation learning were generally positive, none of the studies considered the educators' perspectives. This gap in information is crucial because educators are essential stakeholders in medical education and their opinion and perception of telehealth can shape their educational approaches.

The COVID‐19 outbreak limited face‐to‐face encounters with patients,^33^ and online learning was often offered as an alternative. While students perceived online learning as insufficient to gain clinical skills,^34^ reports on adapted placements to ensure patient encounters during the pandemic were published.^17^, ^35^ The exposure to teleconsultations increased the perceived likelihood that students would consider using teleconsultation in the future. The extent to which students felt confident in using teleconsultation, however, was not considered by the studies included in this review. As most studies reported that students with an interest in telehealth in general elected to participate in this learning, they may have held prior positive attitudes to the topic of teleconsultation. It is therefore difficult to fully determine the impact on their learning,^17^ especially because most studies utilised self‐reported data rather than performance‐based direct measures. Despite recent findings on virtual communication among medical students, which indicated that video consultations with simulated patients reduced nervousness and increased confidence levels in skills required to conduct videoconsultations,^36^ early exposure is limited in health care and social work education. Even though ICT are used daily by most people,^37^ the use of technology and tools in teleconsultations might be unknown to students.^38^

None of the studies described training for social work students, and limited evidence was reported in relation to undergraduate allied health professional programmes.^25^, ^30^ Therefore, most of the results relate to teleconsultation courses in medical education. This limitation highlights the need for research to explore a wider range of experiences and perspectives within health and social work. Insight into a broader range of health and social work students' perceptions and experiences would illuminate the enablers and barriers to providing teleconsultation courses in those disciplines.

While there were limited reports of educators' perspectives, their opinions on teleconsultation education revealed important insights in their willingness and perceived preparedness to teach and facilitate students' learning.^39^ Wentink et al.^40^ highlighted the discrepancy between educators who are familiar with and confident in using eHealth tools and educators who did not have these competencies. This can result in barriers, uncertainties and less positive opinions of teaching telehealth^40^ and suggests that training for educators could improve confidence and increase student uptake of teleconsultation courses at undergraduate level.

As determined by the results of the critical appraisal, it should be noted that there were some limitations in terms of the quality of some of the studies. The research question, research methodology and results of some of the qualitative studies were often not aligned. Moreover, results often did not represent the participants' opinions adequately, which lead to dissonant conclusions and interpretations of the data. Quantitative research designs rarely used reliable, valid measurement tools to collect data and failed to acknowledge confounding factors.

Three limitations are acknowledged in this systematic review. Firstly, it is limited by the quality of eligible studies. Further research involving changes in knowledge and the educators' perspective are warranted to fully understand the effectiveness of undergraduate education in telehealth. Secondly, the search term ‘allied health students’ might have led to an unintentional exclusion of relevant papers focussing on individual professional groups within the allied health professions. Thirdly, the strict limitations to teleconsultation did not report on education in the broader realm of telehealth. Including general training in telehealth without a necessary connection to teleconsultation could have resulted in a greater number of studies.

## 5 CONCLUSION

As the use of telehealth increases, it has become more important to educate and prepare the future workforce. This review summarised current approaches in teleconsultation education for undergraduate health care and social work students. Attention must be drawn to the limited evidence to guide how this education could be implemented in health care and social work education and how effective this education is and its impact on healthcare practice in general as well as interprofessional collaborative practice. More international, interprofessional research on effective implementation of teleconsultation education in undergraduate health care and social work programmes is warranted. The diverse and emerging nature of teleconsultation education suggests that the development of a standard curriculum, informed by the findings of this review and the views of educators and students, may benefit the sector more broadly.

## CONFLICT OF INTEREST

The authors have no conflict of interest to disclose.

## ETHICAL APPROVAL

An ethics approval is not necessary because it is a literature review only with no animals or humans involved.

## Supporting information


Supporting Information S1
