# Supplementary material for: Teleconsultation in health and social care professions education: A systematic review
Source: Clin Teach. 2022 Jul 27;19(5):e13519. doi: 10.1111/tct.13519 (PMC9544545; doi:10.1111/tct.13519)
Supplement: Supplementary file 1 — Supporting Information S1 [file TCT-19-0-s001.docx]

# Supplementary files

## Supplement A: Example for search strategy in PubMed

| S | Setting  *where?* | medical schools, schools of medicine, medical university,  health care and social work institute, university of applied health science, college of nursing, undergraduate health profession education | ((((((((medic*[Title/Abstract] AND school[Title/Abstract]) OR (medic*[Title/Abstract] AND university[Title/Abstract])) OR (health science*[Title/Abstract] AND university[Title/Abstract])) OR (social care[Title/Abstract] AND university[Title/Abstract])) OR (allied health[Title/Abstract] AND university[Title/Abstract])) OR (allied health[Title/Abstract] AND college[Title/Abstract])) OR (allied health[Title/Abstract] AND school[Title/Abstract])) OR (health science*[Title/Abstract] AND school[Title/Abstract])) OR (health science*[Title/Abstract] AND college[Title/Abstract])  Since 2005  109,667 results |
| --- | --- | --- | --- |
| P | Perspective  *for whom?* | undergraduate medical student, undergraduate health care students, undergraduate allied health student, social work student  teacher, professor, trainer, educator, tutor, facilitator | (undergraduate[Title/Abstract]) AND (student*[Title/Abstract]) since 2005  21,104 results  (((((teacher[Title/Abstract]) OR (professor[Title/Abstract])) OR (tutor[Title/Abstract])) OR (facilitator[Title/Abstract])) OR (educator[Title/Abstract])) OR (trainer[Title/Abstract])  Since 2005  34,093 results |
| I | Intervention  *what?* | pre-registration learning programme, training, teaching, learning, seminar, lecture, workshop,  teleconsultation, video consultation, remote communication, telecommunication, information communication technology | ((((((pre-registration[Title/Abstract]) OR (training[Title/Abstract])) OR (lesson[Title/Abstract])) OR (lecture[Title/Abstract])) OR (seminar[Title/Abstract])) OR (workshop[Title/Abstract])) OR (learning[Title/Abstract])  532,603 results  (((remote* AND communication) OR (remote* consultation)) OR (information communication technolog*)) OR ((teleconsultation[Title/Abstract]) OR (telecommunication[Title/Abstract])) OR (video consultation[Title/Abstract]))  37,474 results |
| C | Comparison  *compared with what?* | face to face communication, face to face consultation, in person communication, in person consultation |  |
| E | Evaluation  *with what result?* | skills gained, knowledge, eHealth literacy  attitudes, experience, feedback, recommendation, thought, challenge, difference | ((skills[Title/Abstract]) OR (knowledge[Title/Abstract])) OR (eHealth[Title/Abstract] AND literacy[Title/Abstract])  637,074 results  (((((((attitude[Title]) OR (experience[Title])) OR (opinion[Title])) OR (thought[Title])) OR (feedback[Title])) OR (recommendation[Title])) OR (challenge[Title])) OR (difference[Title])  178,632 results |

| **Search string** | **Results** | **Focus areas** |
| --- | --- | --- |
| ((((undergraduate[Title/Abstract]) AND (student*[Title/Abstract])) AND (((((((pre-registration[Title/Abstract]) OR (training[Title/Abstract])) OR (lesson[Title/Abstract])) OR (lectur[Title/Abstract])) OR (seminar[Title/Abstract])) OR (workshop[Title/Abstract])) OR (learning[Title/Abstract]))) AND ((((remote* AND communication) OR (remote* consultation)) OR (information communication technolog*)) OR ((teleconsultation[Title/Abstract]) OR (telecommunication[Title/Abstract])) OR (video consultation[Title/Abstract])) AND (2005:2021[pdat]))) AND (((skills[Title/Abstract]) OR (knowledge[Title/Abstract])) OR (eHealth[Title/Abstract] AND literacy[Title/Abstract])) | 76 results | Students  Skills |
| ((((undergraduate[Title/Abstract]) AND (student*[Title/Abstract])) AND (((((((pre-registration[Title/Abstract]) OR (training[Title/Abstract])) OR (lesson[Title/Abstract])) OR (lecture[Title/Abstract])) OR (seminar[Title/Abstract])) OR (workshop[Title/Abstract])) OR (learning[Title/Abstract]))) AND ((((remote* AND communication) OR (remote* consultation)) OR (information communication technolog*)) OR ((teleconsultation[Title/Abstract]) OR (telecommunication[Title/Abstract])) OR (video consultation[Title/Abstract])))) AND ((((((((attitude[Title]) OR (experience[Title])) OR (opinion[Title])) OR (thought[Title])) OR (feedback[Title])) OR (recommendation[Title])) OR (challenge[Title])) OR (difference[Title])) | 3 results | Students  Attitudes |
| (((((((((social work) AND (school)) OR (university)) OR (college)) OR ((((applied health science) AND (school)) OR (university)) OR (college))) OR ((((medic*) AND (school)) OR (university)) OR (college))) AND ((((((teacher[Title/Abstract]) OR (professor[Title/Abstract])) OR (tutor[Title/Abstract])) OR (facilitator[Title/Abstract])) OR (educator[Title/Abstract])) OR (trainer[Title/Abstract]))) AND (((((((pre-registration[Title/Abstract]) OR (training[Title/Abstract])) OR (lesson[Title/Abstract])) OR (lecture[Title/Abstract])) OR (seminar[Title/Abstract])) OR (workshop[Title/Abstract])) OR (learning[Title/Abstract]))) AND ((((remote* AND communication) OR (remote* consultation)) OR (information communication technolog*)) OR ((teleconsultation[Title/Abstract]) OR (telecommunication[Title/Abstract])) OR (video consultation[Title/Abstract])) AND (2005:2021[pdat]))) AND (((skills[Title/Abstract]) OR (knowledge[Title/Abstract])) OR (eHealth[Title/Abstract] AND literacy[Title/Abstract])) | 65 results | Teacher  Skills |
| ((((((((teacher[Title/Abstract]) OR (professor[Title/Abstract])) OR (tutor[Title/Abstract])) OR (facilitator[Title/Abstract])) OR (educator[Title/Abstract])) OR (trainer[Title/Abstract])) AND (((((((pre-registration[Title/Abstract]) OR (training[Title/Abstract])) OR (lesson[Title/Abstract])) OR (lecture[Title/Abstract])) OR (seminar[Title/Abstract])) OR (workshop[Title/Abstract])) OR (learning[Title/Abstract]))) AND ((((remote* AND communication) OR (remote* consultation)) OR (information communication technolog*)) OR ((teleconsultation[Title/Abstract]) OR (telecommunication[Title/Abstract])) OR (video consultation[Title/Abstract])) AND (2005:2021[pdat]))) AND ((((((((attitude[Title]) OR (experience[Title])) OR (opinion[Title])) OR (thought[Title])) OR (feedback[Title])) OR (recommendation[Title])) OR (challenge[Title])) OR (difference[Title]) AND (2004:2021[pdat])) | 7 results | Teacher  Attitudes |

## Supplement B: Critical appraisal tools and outcome

| **Types of studies** | **Assessment** |
| --- | --- |
| Qualitative studies | **JBI Critical Appraisal Checklist for Qualitative Studies**  **JBI Qualitative studies** |
| Randomised controlled trials | **RoB 2** |
| Non-randomised controlled trials | **JBI Critical Appraisal Checklist for Quasi-Experimental Studies (non-randomized experimental studies)** |
| Cross-sectional design | **JBI Critical Appraisal Checklist for Analytical Cross-Sectional Studies** |
| Case reports | **JBI Critical appraisal Checklist for Case Reports** |
| Cohort studies | **JBI Critical appraisal checklist for cohort studies** |
| Case-control studies | **JBI Critical appraisal checklist for case-control studies** |

Table 3: Critical appraisal tools used for assessing the quality of included studies.

| **Study** | **C1** | **C2** | **C3** | **C4** | **C5** | **C6** | **C7** | **C8** | **C9** | **C10** |
| --- | --- | --- | --- | --- | --- | --- | --- | --- | --- | --- |
| Abraham et al., 2020 (Qual) | N | N | N | N | N | N | N | N | N | N |
| Abraham et al., 2020 (Quant) | N | Y | NA | NA | N | N | N | N | / | / |
| Bulik et al., 2010 (Qual) | N | N | U | N | N | N | N | U | Y | N |
| Bulik et al., 2010 (Quant) | Y | U | N | U | N | N | U | N | / | / |
| Cantone et al., 2019 | N | N | N | N | N | N | N | U | Y | U |
| Christner et al., 2010 | N | N | N | Y | N | Y | N | Y | / | / |
| Darnton et al., 2020 | U | U | U | Y | Y | U | Y | Y | Y | Y |
| Dzara et al., 2013 | U | U | U | N | U | N | U | Y | / | / |
| Edirippulige et al., 2012 | N | Y | N | N | N | N | N | N | NA | N |
| Fisher et al., 2014 (qual) | N | N | U | U | U | N | N | N | N | N |
| Fisher et al., 2014 (quant) | U | Y | U | U | N | N | N | Y | / | / |
| Jimenez-Rodriguez & Arrogante, 2020 (qual) | U | U | U | U | U | U | N | Y | Y | U |
| Jimenez-Rodriguez & Arrogante, 2020 (quant) | Y | Y | Y | U | N | N | Y | Y | / | / |
| Mulcare et al., 2020 (Qual) | U | U | Y | N | N | N | N | N | Y | N |
| Mulcare et al., 2020 (Quant) | NA | NA | NA | N | Y | U | N | U | U | / |
| Palmer et al., 2015 | N | Y | U | N | N | N | N | N | Y | N |
| Palmer et al., 2017 | Y | U | Y | N | N | N | Y | U | Y | / |
| Rientis et al., 2016 (Qual) | N | N | N | N | N | N | N | N | N | N |
| Rientis et al., 2016 (Quant) | Y | N | N | N | N | N | N | U | U | / |
| Waschkau et al., 2020 (Qual) | N | N | U | N | N | N | N | N | N | N |
| Waschkau et al., 2020 (Quant) | Y | Y | N | NA | N | N | U | N | / | / |

Table 4: Outcome of the critical appraisal ordered by the criteria 1 (C1) to criteria 10 (C10).
N (no), Y (yes), NA (not applicable), U (unclear), Qual (qualitative part of the mixed-methods study), Quant (quantitative part of the mixed methods study).

## Supplement C: PRISMA Checklist

| **Section/topic** | **#** | **Checklist item** | **Reported on page #** |
| --- | --- | --- | --- |
| **TITLE** | | |  |
| Title | 1 | Identify the report as a systematic review, meta-analysis, or both. | 1 |
| **ABSTRACT** | | |  |
| Structured summary | 2 | Provide a structured summary including, as applicable: background; objectives; data sources; study eligibility criteria, participants, and interventions; study appraisal and synthesis methods; results; limitations; conclusions and implications of key findings; systematic review registration number. | 1 |
| **INTRODUCTION** | | |  |
| Rationale | 3 | Describe the rationale for the review in the context of what is already known. | 2 |
| Objectives | 4 | Provide an explicit statement of questions being addressed with reference to participants, interventions, comparisons, outcomes, and study design (PICOS). | 2 |
| **METHODS** | | |  |
| Protocol and registration | 5 | Indicate if a review protocol exists, if and where it can be accessed (e.g., Web address), and, if available, provide registration information including registration number. | POSPERSO ID CRD42020220883 |
| Eligibility criteria | 6 | Specify study characteristics (e.g., PICOS, length of follow-up) and report characteristics (e.g., years considered, language, publication status) used as criteria for eligibility, giving rationale. | 3 |
| Information sources | 7 | Describe all information sources (e.g., databases with dates of coverage, contact with study authors to identify additional studies) in the search and date last searched. | 3 |
| Search | 8 | Present full electronic search strategy for at least one database, including any limits used, such that it could be repeated. | Supplement A |
| Study selection | 9 | State the process for selecting studies (i.e., screening, eligibility, included in systematic review, and, if applicable, included in the meta-analysis). | 3, Supplement |
| Data collection process | 10 | Describe method of data extraction from reports (e.g., piloted forms, independently, in duplicate) and any processes for obtaining and confirming data from investigators. | 3 |
| Data items | 11 | List and define all variables for which data were sought (e.g., PICOS, funding sources) and any assumptions and simplifications made. | Table 1 |
| Risk of bias in individual studies | 12 | Describe methods used for assessing risk of bias of individual studies (including specification of whether this was done at the study or outcome level), and how this information is to be used in any data synthesis. | Supplement B |
| Summary measures | 13 | State the principal summary measures (e.g., risk ratio, difference in means). | - |
| Synthesis of results | 14 | Describe the methods of handling data and combining results of studies, if done, including measures of consistency (e.g., I^2^) for each meta-analysis. | 3 |
| Risk of bias across studies | 15 | Specify any assessment of risk of bias that may affect the cumulative evidence (e.g., publication bias, selective reporting within studies). | - |
| Additional analyses | 16 | Describe methods of additional analyses (e.g., sensitivity or subgroup analyses, meta-regression), if done, indicating which were pre-specified. | - |
| **RESULTS** | | |  |
| Study selection | 17 | Give numbers of studies screened, assessed for eligibility, and included in the review, with reasons for exclusions at each stage, ideally with a flow diagram. | 3 |
| Study characteristics | 18 | For each study, present characteristics for which data were extracted (e.g., study size, PICOS, follow-up period) and provide the citations. | Table 2 |
| Risk of bias within studies | 19 | Present data on risk of bias of each study and, if available, any outcome level assessment (see item 12). | Supplement B |
| Results of individual studies | 20 | For all outcomes considered (benefits or harms), present, for each study: (a) simple summary data for each intervention group (b) effect estimates and confidence intervals, ideally with a forest plot. | - |
| Synthesis of results | 21 | Present results of each meta-analysis done, including confidence intervals and measures of consistency. | - |
| Risk of bias across studies | 22 | Present results of any assessment of risk of bias across studies (see Item 15). | - |
| Additional analysis | 23 | Give results of additional analyses, if done (e.g., sensitivity or subgroup analyses, meta-regression [see Item 16]). | - |
| **DISCUSSION** | | |  |
| Summary of evidence | 24 | Summarize the main findings including the strength of evidence for each main outcome; consider their relevance to key groups (e.g., healthcare providers, users, and policy makers). | 6-8 |
| Limitations | 25 | Discuss limitations at study and outcome level (e.g., risk of bias), and at review-level (e.g., incomplete retrieval of identified research, reporting bias). | 9 |
| Conclusions | 26 | Provide a general interpretation of the results in the context of other evidence, and implications for future research. | 9 |
| **FUNDING** | | |  |
| Funding | 27 | Describe sources of funding for the systematic review and other support (e.g., supply of data); role of funders for the systematic review. | 1 (non-anonymised file) |

*From:*  Moher D, Liberati A, Tetzlaff J, Altman DG, The PRISMA Group (2009). Preferred Reporting Items for Systematic Reviews and Meta-Analyses: The PRISMA Statement. PLoS Med 6(7): e1000097. doi:10.1371/journal.pmed1000097

For more information, visit: **www.prisma-statement.org**.

Page 2 of 2

## Supplement D: Eligibility criteria

| **Inclusion** | **Exclusion** |
| --- | --- |
| **Journal requirements / Study designs** | |
| Published in peer reviewed journals | Published before 2010 |
| Published in English, German, or Danish | Systematic reviews |
| Experimental and quasi-experimental studies are accepted in this review. These include non-randomised and randomised controlled trials, before-after-designs | Grey literature (e.g. policy papers, unpublished resources, informal, opinion letters, conference reviews) |
| Qualitative study designs including interviews, focus groups, observations, and case studies are eligible |  |
| **Population / Perspective** | |
| Evidences must address undergraduate medical students, health care students and/or social work students | Training sessions that include qualified professionals |
| **Intervention** | |
| Training sessions must educate students in using and participating in telecommunication and teleconsultation and/or provide relevant context information (e.g. medio-legal information, ethics) | Training sessions that focus only on educating the use of other telemedical technologies (e.g. personal health records, telesurgery tools) |
| Training sessions can be delivered virtually and non-virtually | Studies focussing on digital learning (e-learning) with no connection to telecommunication and teleconsultation (i.e. using technologies for delivering information to students) |
| Studies can compare training sessions for virtual and non-virtual communication and consultation | Evidences that investigate general opinions on and perceptions of telemedicine |
| Evidences must evaluate the skills and/or knowledge about telecommunication and teleconsultation gained throughout a training session | Articles describing patient education |
| Studies evaluating the opinions and feedback (perception, experiences, attitudes) given from medical, nursing, and allied health professionals, and social work students and/or educators regarding training sessions |  |

Table 5: Eligibility criteria based on the SPICE framework

## Supplement E: Codes developed from the qualitative data

| Author | Year | Qotations | Codes |
| --- | --- | --- | --- |
| Bulik | 2010 | “The combination of these experiences was very educational and inspiring. In fact, I found telemedicine to be so useful and beneficial to the underserved population that I became interested in making telemedicine part of my career as a pediatric cardiologist.” | Benefits for patients, positive feedback, future use |
|  |  | “Exposure has been the best teacher for me over the past four decades of my life. It was an awesome experience to see telemedicine in action, as opposed to the two-minute attraction of passing by it to impress students who are interviewing.” | positive feedback, exposure to TM |
|  |  | “In summary, this course greatly opened my eyes to the vast possibilities that telemedicine has to offer.” | positive feedback |
|  |  | “These last four weeks have been a unique educational experience for me. I had heard much about telemedicine and its growth, but did not have the opportunity to see it in play, from both the provider and patient perspective. I am especially interested in telemedicine because I wished a part of my time as a health care provider to be directed toward the underserved population, but am reluctant to live in or commute to a rural clinic.” | Benefits for patients, positive feedback |
|  |  | “Following my one-month elective in telemedicine at UTMB, I am excited and look forward to incorporating electronic health into my medical practice. | future use |
|  |  | “(This university) is the largest telemedicine provider in the state … but the medical students are not getting any exposure to it. None of our third-year clinical rotations requires us to see even one session of telemedicine, and this should change.” | telemedicine in educational setting |
|  |  | As for me personally, I will begin a Pathology residency program…so I see it fitting to address telepathology and how it will affect my practice in the future… There are also applications of telepathology for the autopsy specialist. If I embark on an anatomic pathology career in performing autopsies, I may not be fully trained to perform a difficult fetal autopsy. This would require 3D image to be taken of the representative subject and sent for consultation, ultimately via telepathology. An important pitfall with remote processing of images to pathologists is that the image being viewed is only a computerized representation of reality and therefore the eye should be trained to look at these types of images before making a diagnosis. Overall, as you can surmise from the details above, the advantages of telemedicine to the field of pathology in particular far outweigh any disadvantages; thus I am confident I will be utilizing this technology in my future pathology career. | future use, benefits of TM |
|  |  | “As a graduating 4th year medical student…I have been underexposed to methods of providing indigent care outside of the major university clinics. My experiences interacting with the telemedicine physicians, nurses, and patients has opened my eyes to a method of healthcare delivery that is not typically taught to medical students. I will be starting a residency in Physical Medicine and Rehabilitation in the near future. Challenges in this particular field include the chronicity of injuries, the expense and labor involved in caring for this subset of patients, the lack of self-sufficiency that many of the patients have, and the broad scope of patients and patients’ issues that are dealt with. The scope of patients can include everything from workers compensation cases, to geriatric health, to severe brain injury. All of these particular challenges can be addressed effectively through telehealth. Telemedicine can allow a provider to perform follow-up examinations and routine follow-up care from a distance. In this patient population, it is especially difficult for the patient to commute to the physician’s office….From my experiences in the clinics, patients appreciate the accessibility of the provider, as well as the reassurance that everything is going well.” | benefits for patients, future use |
| Cantone | 2019 | “It was a good experience working with telemedicine.” | positive feedback |
|  |  | “I liked the telemedicine session as that was a good opportunity for us to get our feet wet in a no-stakes environment!” | positive feedback, exposure to TM |
|  |  | “I enjoyed the telemedicine station where we had to figure it out on the fly.” | positive feeback, exposure to TM, problem solving |
|  |  | “Helpful tips in using technology and telemedicine concerns.” | positive feeback, problem solving |
|  |  | “I enjoyed being able to practice telemedicine, I think it would be a valuable experience for everyone to try.” | positive feeback, exposure to TM |
|  |  | “More instruction or training on how best to practice telemedicine would be a welcome addition.” | more training required |
|  |  | “It would be nice to have some sort of orientation to TeleOSCE and the software before being thrown in. I felt it was a stressful experience.” | more training before |
|  |  | Telemedicine is a unique opportunity to cross multiple barriers, including cost and distance, to patient care. | benefits for patients, benefits of TM |
|  |  | The telemedicine OSCE taught me . . . how much it can cost, both in dollars and time, for patients in rural areas to go to the hospital, clinic, and/or pharmacy.” | benefits for patients, benefits of TM |
|  |  | “Telemedicine seems like a useful healthcare tool that, in the right situations, might help strike a balance between value and cost.” | benefits of TM |
|  |  | Telemedicine can be an effective method to bring value to patients who live far from a clinic/area with shortage of physicians for less cost. | benefits for patients, benefits of TM |
|  |  | “Excellent opportunity to use a technology I had never had a chance to use before.” | exposure to TM and technology |
|  |  | “Very useful in seeing how telemedicine is practiced! It was nice to have this experience before actually trying telemedicine in the real world.” | exposure to TM, future use |
|  |  | “It was a difficult medium to do a patient exam but I can see the usefulness in reaching remote patients with no other access to care.” | benefits for patients, physical exam, exposure to TM |
|  |  | Challenging as well as informative for using the technology.” | expsosure to TM and technology |
|  |  | I do appreciate that remote discussions with providers can give enough info if the patient is a good historian, but I miss the personal connection. Building a trusting relationship with a patient may be harder.” | negative aspects of TM |
|  |  | “It taught me how to assess triaging in a virtual setting. It allows me to be able to talk with patients and focus on building a rapport in a novel medium. | expsosure to TM and technology, skills |
|  |  | This experience cautioned me to be more aware of my internal bias toward in-person visits vs telemedicine calls.” | learning outcome, attitude |
|  |  | Maybe a slightly simpler case for students ...so that they can focus more on the technology.” | critique on training session, expsosure to TM and technology |
|  |  | “I see that telemedicine can be a way to overcome a barrier to health care, but I found it difficult to provide quality care.” | negative aspects of TM |
|  |  | “I think that the telemedicine OSCE was really tricky. I definitely appreciate the use of this technology for accessing more rural communities or people who could not otherwise access care. However, I felt like it was a slower and more cumbersome process for the provider and I definitely felt uncomfortable not being able to examine the patient.” | exposure to TM and technology, benefits for patients, physical examination |
|  |  | Was unsure whether we were allowed to click on computer. Would have appreciated one line on the prompt sheet that says that the computer is ‘in play.’” | critique on training session |
|  |  | Tell students that we are being evaluated on HOW we use the technology—we thought it was more about how we took the history, etc., but necessarily knowing that we were being critiqued on how we utilized telemedicine.” | critique on training session, expsosure to TM and technology |
|  |  | “It helped me understand that tele-encounters are done out of necessity and unfortunately have several drawbacks.” | negative aspects of TM |
|  |  | Again, it was useful to practice using telemedicine in a low stakes environment. It was pretty awkward so having that exposure was really helpful.” | exposure to TM, comfortness |
|  |  | “It wasn’t enough time to practice telemedicine skills, nor was there enough context for it to be a very effective educational experience.” | critique on training session |
|  |  | “I think more time needs to be allotted due to unforeseen technological issues.” | critique on training session, technical problems |
|  |  | “A little more time to work with the system/read the case before the patient was online would have been appreciated.” | critique on training session, patient case |
|  |  | “I am not yet entirely convinced about telemedicine and getting the same value out of this visit versus the telemedicine visit. It is more difficult to develop a strong physician-patient relationship, so I think I need a bit more experience to understand the value and trade-offs for telemedicine.” | critique on training session, future use, negative aspects of TM |
|  |  | “Appreciate that it can be used as a triage function and save families difficult or time consuming/gas consuming trips.” | benefits of TM, benefits for patients |
| Darnton | 2020 | ‘I think it was quite time consuming selecting the patients. You know, the GP selecting the patients, because every time we called them to book them, you would almost do a mini consult in advance’ | challenges of TM |
|  |  | ‘We had to just pre-select the right patients. So, from a list of, sort of, I don’t know, in the morning, kind of, 80–100 patients just finding the ones that were the most value.’ | challenges of training sessions, chosing the right patient |
|  |  | ‘I tried to get …patients that we knew but that we hadn’t seen before that were presenting with acute stuff so that the students could take a nice fresh history’ | challenges of training sessions, chosing the right patient |
|  |  | We, actually, cherry picked the patients from a little bit earlier on in the week or the previous week and just asked them to tell their story as they had to us.’ | challenges of training sessions, chosing patients |
|  |  | ‘so the first one was a star patient because I’d tried five different people …So, then I thought I’m just going to call this lady who I’ve been seeing loads recently, who has an interesting history …’ | challenges of training sessions, chosing patients |
|  |  | ‘my colleague said … that one of the students did seem to be distracted, and looking sideways during the video. … So I think he [the supervisor] stopped it and said is everything alright, is your environment okay, shall we bring you back in for the next consultation? And he said yeah …, I think there was definitely some kind of noise distraction that was affecting his ability to consult. … and so his colleague took over… and he responded well to the feedback, and there weren’t any further issues.’ | issues during consultations |
|  |  | [fear of] ‘… family members just like storming in through the door, although I did warn them that I was going to do our meeting consultation … you never know if they will forget. So if someone’s in the back of your mind, like, oh, if you hear a noise or something, that can distract you sometimes from the consultation.’ | challenges of TM, issues during consultations |
|  |  | ‘So there were times when the technical difficulties…they were kind of looking to me, like what should we do. So, I did have to dip in a few times, oh let’s try this, or let’s try that. But it didn’t take away from the flow I don’t think. It seemed okay.’ | challenges during consultations, challenges of TM, technical issues |
|  |  | ‘…both of us felt that they saw it wasn’t working and watched to see what we would do. Which is interesting, but we can’t do much because we didn’t set up the consultation and we don’t have access to the Teams to set up for a patient, and it wouldn’t be appropriate to anyway. So, I think maybe if they could step in a little bit sooner if there was a problem with that would be useful.’ | technical issues, feedback to students |
|  |  | ‘Like I, sort of, thought maybe the GP would just jump in once there was technical issues, but they, kind of, hung back so it was, kind of, us that were like trying to reassure the patient and like trying to see if that connection…if the technical connection was there. I think, that was useful because, I think, it’s going to be more of a skill going forwards, I guess. Yes, learning how to handle that was, yes, it was useful’ | technical issues, skills, learning outcomes |
|  |  | I could ask questions after or before each patient consultation, but I felt like there wasn’t as much time spent with the tutor. Because obviously when you’re in the practice in person you have lunch breaks and coffee breaks and you can discuss cases and ask questions then.’ | challenges of TM, relationship to educators |
|  |  | I was probably more inclined to be more neutral … I guess normally when they’re in, they would feel more comfortable, you would be more comfortable, you’d ask a bit more about their life, and how they got here, and how they were. And you lose all that in a video, it’s very much down to business immediately. So I guess with that, you’ve got slightly less rapport, it’s harder to give feedback. Or feedback can be taken in the wrong way, or taken more personally, I think, by video. Whereas when they’re in the room with you, you can kind of understand their…whether they’ve got it, and how they respond immediately, in a much clearer way, I would say, yeah | comfortness, relationship to educators |
|  |  | in the surgery … if the student was talking to me, she would have had to turn herself, physically turn herself slightly away from the patient and not make eye contact with the patient anymore. Whereas when you’re doing it on a screen you’re almost making eye contact with everybody at the same time because you’re looking into the camera. So it’s a lot more difficult to shift the focus from student talking to patient to student talking to tutor, I think.’ | nonverbal communication, relationship to educators |
|  |  | It’s just the before and after part really, the ease and the … clunkiness … in terms of actually having the pre and post-consultation discussion, really. …we were sort of essentially talking expecting the patient to join the meeting at any time so you’re really just quickly squeezing a few words in and it just felt a little bit rushed. Whereas … say the patient had arrived for a face to face, then whetheryou take an extra minute ortwo or three orfourhaving a pre consultation discussion doesn’t matter too much and then you choose to then say, I’m going to go and collect the patient.’ | challenges of TM, communication with patients |
|  |  | ‘I think it’s not as bad as…yes, as it would be being in a ward. Because a lot of it is down to taking a history …’ | feedback on training session |
|  |  | I think it was good experience to do video consultations, especially as it might be going towards that in the future.’ | positive feedback |
|  |  | … having to adjust to asking questions that you would have found out the answers for in an exam, but asking the patient the questions instead, which is good I think practical medical experience …’ | communication with patients |
|  |  | ‘They wanted to talk to us. I think sometimes in a GP [primary care placement] as well when the patient comes into the room and they see student doctors, and the doctor just says, oh is it okay if they’re here, they kind of have to say yes. I mean, they can say no, but probably sometimes the pressure will cause them to just be like, oh yeah it’s fine. But they have to formally consent so the GP rang them before speaking to us in the same room and so it was like they actually wanted to speak to us.’ | communication with patients, chosing the patients |
| Eddirippulige | 2012 | I learned how to set up a videoconference. | skills, technical skills |
|  |  | Knowing the differential use of bandwidth is useful. | technical skills |
|  |  | I learned how to take clinically appropriate pictures. | technical skills |
|  |  | I would like to use these skills when I become a doctor. I realized how ICT can be used for my consultations. | future use, skills |
|  |  | My teaching profession can benefit from some of these skills. | skills |
|  |  | I can use these skills in my profession as a clinical pharmacist working in rural areas. | skills, future use |
|  |  | I learned how to use technology, particularly videoconferencing, for clinical practice. | future use, technical skills |
|  |  | Possibly I can help doctors and patients from developing countries. | future use |
|  |  | As a speech pathology specialist in rural/remote location, eHealth techniques can be very useful. | benefits for patients |
|  |  | eHealth skills will be useful as a physiotherapy professional. | skills |
|  |  | This will be useful in home monitoring of rehab programs. | future use |
|  |  | As a medical professional, eHealth tools will be very useful in the future. | future use |
|  |  | eHealth can be useful in maintaining contacts with clinicians. | relationship with patients, communication with patients |
|  |  | The skills acquired in the practicum will be helpful to assist my patients to reduce travel and save money | skills, future use |
|  |  | These skills can help patients, especially those who do not have access to allied health services in rural areas. | benefits for patients |
|  |  | I will use videoconferencing in my psychology advice to provide services to remote patients. | future use |
|  |  | I realized that simple technologies such as webcams can be used to consult patients in regional areas (where mental health may not be accessible). | benefits for patients, technical skills, knowledge |
|  |  | eHealth can be a useful tool in aged care. | benefits for patients |
|  |  | The practicum helped me become more aware of the direction technology is shaping health care. | exposure to TM and technology |
|  |  | Simple webcams can be a useful tool to help patients. | exposure to TM and technology |
| Fisher | 2014 | ‘It was useful that colleagues could hear and learn from everyone ’ s attempts’ | positive feedback |
|  |  | ‘Good practise at interprofessional communication – would like more!’ | communication skills |
|  |  | ‘Highlighted areas of defi cit in my communication skills’ | practice communication skills |
|  |  | ‘Learnt about structuring questions to make sure I got the appropriate clinical information’ | practice communication skills |
|  |  | ‘Controlled, but safe, way of feeling like a doctor’ | positive feedback |
|  |  | ‘Really good! Felt like doing “real” stuff!’ | positive feeback |
|  |  | ‘Good practise at how to phrase questions when the cause for the phone call not immediately apparent’ | practice communication skills |
|  |  | ‘Really good – there ’ s been no other opportunities to practise stuff like this’ | exposure to TM, positive feedback |
| Jimenez | 2020 | I liked it even more than I thought I would have, and I have learned and enjoyed it a lot” | skills, positive feedback |
|  |  | I had fun seeing how my peers acted, thinking about what I would have done and what should be corrected, so my experience has been satisfactory, and I’ve learned not only the theory to address, but also attitudes and how to manage a situation that I may not have experienced before: I have gained resources and knowledge” | skills, attitudes, positive feedback |
|  |  | A complete novelty, of which I’m grateful to have been a part of, as they had never spoken to us about this possibility as an interview method and to be in contact with patients, so that I am grateful in light of the future” | future use, exposure to TM, positive feedback |
|  |  | It has been a good alternative in light of the current situation we are facing” | positive feedback, COVID 19 |
|  |  | Realism, correcting mistakes, empathy, improvement of attitudes and skills, trust, not being embarrassed anymore, non-verbal language techniques. Promoting group participation” | attitudes, skills, non verbal communication |
|  |  | Learn how telehealth will be, as it is something that is being utilized in the health centers due to the current situation we are in, and I think it is important to know how to create a good environment for the patient in distance health services, because sometimes it is complicated” | COVID 19, exposure to TM, benefits for patients |
|  |  | I have learned new communication skills, to pay attention to the verbal and non-verbal language, to listen to their worries and solve doubts, to explain myselfusing an easy and clear language, to create comfortable surroundings, and most of all to relax myselfand deal directly with the patient. This has allowed me to have an idea about how to manage possible situations that I may experience with real future patients” | practice communication skills, non verbal communication, comfortness, future use |
|  |  | It is an approach to a real situation with many realistic issues, it provides options for developing communication and psychological techniques, it lets us learn from our mistakes before making them in real life, and it makes you become truly involved with the patient, searching for all the means to help him or her” | practice communication skills, positive feedback |
|  |  | You feel less nervous when you are performing the simulation from your house and within your comfort zone” | comfortness, working from home |
|  |  | I think we have felt less embarrassed and with more confidence when performing the simulation through the screen” | comfortness, confidence |
|  |  | When being in my own room, it didn’t feel like an exam, and this has resulted in me being more calm | comfortness |
|  |  | “Less nervousness” | comfortness |
|  |  | Maybe the main problem was the internet connection, but this is not dependent on the simulation” | technical issues |
|  |  | Maybe the quality of the connection, but there isn’t much to improve” | technical issues |
|  |  | The connection problems (could be a positive issue ifyou know how to deal with it” | technical issues |
|  |  | Due to the current situation of having to do the simulation online, practical skills have not been able to be demonstrated | COVID 19, challenges in education |
|  |  | We have not been able to improve the technical skill in the same way” | technical skills |
|  |  | It’s difficult to learn practical skills with an online simulation, but many other things are learned” | challenges in education |
| Mulcare | 2020 | Clinical application didactics were redundant with previous clerkship learning. | challenges in education, feedback |
|  |  | Too many didactics about telemedicine in general. | critique of training session, content |
|  |  | Unproductive break, time too much. | critque of training session, content |
|  |  | More focus on medical management and appropriate dispositions of patient. | critque of training session, content |
|  |  | Course should take place earlier in the clerkship as opposed to the last week of their clerkship. | critique of training session, time of training |
|  |  | Make the simulation topic a surprise. | critique of training session, patient case |
|  |  | More discussions about where in medicine telemedicine is the most useful. | critique of training session, methods/activities |
|  |  | More details in email prior to course regarding what the course is about so students know what to expect. | critique of training session, preparation |
|  |  | Condense training to 1 day instead of 2 half days. | critique of training session, time of training, content |
|  |  | Give students advanced notice that their homework videos will be shown during the course. | critique of training session, preparation |
|  |  | Review more videos during debrief. | critique of training session, content, methods/activities |
|  |  | Shorter session overall. | critique of training session, time of training, content |
|  |  | Positive feedback in general. | postive feedback |
|  |  | It would be great to see an example telemedicine encounter. | critique of training session, content, methods/activities |
|  |  | Include a recorded physician interview/see example from expert telemedicine attendings. | critique of training session, content, methods/activities |
| Palmer | 2015 | This was a great use of time. It didn't take much time at all, but was an excellent assessment of my knowledge and clinical judgment.’ | positive feedback |
|  |  | I thought this was a really valuable experience.’ | positive feeback, experience |
|  |  | It compares to the OSCEs I've done at [the study institution] other than the fact that it was over a computer and not in person.’ | assessment |
|  |  | I really appreciate not having to drive all the way back to [my home institution] and it worked pretty well.’ | benefits for students |
|  |  | I thought it [the teleOSCE] was very effective. It is kind of a novel way of teaching. Instead of see one, do one, teach one; we just DID one!’ | assessment |
|  |  | ‘I think it [the feedback] was just as good as feedback I have gotten in person.’ | feedback for students, positive feedback |
|  |  | ‘I thought this was a good experience. I think a lot of medical students today have at least cursory experience using online video communication, such as Skype, to communicate with people. This helps integrate technology in an effective way into learning how to care for patients. | positive feedback, exposure to TM and technology |
|  |  | It was nice that the chatroom was in a format that we were familiar with from our other sessions.’ | exposure to technology |
|  |  | ‘Video quality was really clear ... it was really easy to hear and see. It was just like Skyping with someone ... pulling up pictures was doable but harder.’ | exposure to technology, experience with TM |
|  |  | ‘I had issues. When I attempted to open both the sugar log and the photo my laptop first had to download Google Chrome (instead of Internet Explorer I was using) and then download the files. This was just too much, along with the Adobe Connect meeting room up, for my little guy [computer] to pull off all at once. It took nearly 15 minutes before I finally saw the picture. However, it was a good experience because it forced me to make a decision about patient care based solely on hx [patient history] which was good.’ | technical issues, technical problem solving |
|  |  | ‘Before, I thought that telemedicine was mainly for [my home institution] or for big city physicians to kind of consult with rural physicians, you know, like a rural physician would have a patient in their office to, like, consult with a specialist ... but then, after this experience, it kind of taught me that you can actually do visits with patients in their houses. It's never crossed my mind before that patients would have the same technology as the physician in the office so that you could do a visit with the patient in their homes by themselves, like that ... That was new for me.’ | exposure to TM and technology, knowledge |
|  |  | It [the teleOSCE] fits pretty well with the theme that we do have a lot of patients who have a hard time getting in to see the doctor because even though this is a rural area, they live even farther out, so I can definitely see myself doing this, you know, later on in my career when I will have to do telemedicine with patients.’ | assessment, future use, |
|  |  | ‘I think getting used to the idea of telemedicine ... to provide a high level of patient care to their patients is really valuable.’ | positive feedback |
| Waschkau | 2020 | ,,Dass die Themen an sich zur Sprache kamen! Ein guter Anfang und ein Signal, dass sich die Medizin im Wandel befindet.‘‘ | exposure to TM, positive feedback |
